# Nature in Disease; Illustrated by Various Discourses and Essays

**Published:** 1860-07

**Authors:** 


ART. VII.—*Nature in Disease; illustrated by various Discourses and Essays. To which is added Miscellaneous Writings, chiefly on Medical Subjects.* By JACOB BIGELOW, M.D., Professor of Materia Medica in Harvard University, etc. etc. etc. Second edition. 12mo., pp. 410. New York, 1860.

THE fact that a new edition of this work, so soon after the appearance of the first, has been called for, shows the estimation in which it is held by the American profession. The volume has been issued in a very superior manner, and is altogether exceedingly attractive, not only on account of the intrinsic interest of the various topics of which it treats, but the chaste and classical style in which it has been composed. Dr. Bigelow writes like a scholar; and, although we do not feel inclined to indorse all his views, we can most cordially recommend the work as worthy of the most attentive perusal. The discourse on self-limited diseases, originally delivered before the Massachusetts Medical Society, at their annual meeting, in May, 1835, ought to be read and re-read by every member of the profession, as it will afford him a much better idea of the philosophy of treatment in this class of affections than he can find anywhere else within the same compass in the English, or, indeed, in any other language. Dr. Bigelow, in fact, was the first modern physician to call the attention of the profession prominently to the subject, and if he had never written anything else, this discourse alone would justly entitle him to the respect and gratitude of his brethren and of the public. The doctrine of the self-limitation of diseases had, it is true, been occasionally announced by others, among the rest by Asclepiades, the friend of Cicero; but it remained for our Boston confrère to place the subject in its just light, and to infix its importance upon the professional mind. He has shown the folly and wickedness of the use of harsh, violent, and perturbating remedies in the treatment of these maladies, and the indispensable necessity of a proper confidence, on the part of the practitioner, in the resources of nature. All honor, we say, to the man who had the sagacity fully to perceive these inestimable truths, and the courage to announce them to the world. We would rather be the author of such a doctrine than the hero of Waterloo.

Among other topics discussed in this volume are practical views on medical education; the medical profession and quackery; gout and its treatment; aphorisms on cholera; remarks and experiments on pneumothorax; the medicinal effects of coffee and tea; the history and use of tobacco; and the early history of medicine.

The Messrs. Wood have also brought out a new edition of the author's little work, entitled "A Brief Exposition of Rational Medicine," first published about two years ago, and noticed in a former volume of the REVIEW. Whatever may be thought of some of its doctrines, there can be no doubt at all that the book has already done an immense deal of good, by inducing practitioners to have less confidence in their own powers and more in the resources of nature, when properly aided by remedial measures. We heartily recommend an attentive perusal of its pages.

